# Investigation of the spermathecal morphology, reproductive strategy and fate of stored spermatozoa in three important thysanopteran species

**DOI:** 10.1038/s41598-022-23104-0

**Published:** 2022-11-02

**Authors:** Stephanie Krueger, Juliana Martins de S. e Silva, Cristine Santos de Oliveira, Gerald Moritz

**Affiliations:** 1 Institute of Biology, Department Zoology, Martin-Luther University Halle-Wittenberg, Halle, Germany; 2 Institute of Physics, Martin-Luther University Halle-Wittenberg, Halle, Germany; 3 Fraunhofer Institute for Microstructure of Materials and Systems IMWS, Halle, Germany

**Keywords:** Biological techniques, Ecology, Evolution, Physiology, Zoology

## Abstract

In insects, females can keep sperm capable of fertilisation over a long period with the help of the spermatheca. The effectiveness of storing fertile sperm is expected to reflect in the reproductive strategy and, thus, the morphology of the involved organs. In this work, we focused on the relationship between reproduction and morphology in the haplodiploid Thysanoptera, especially if a loss of these traits occurs under thelytoky. The spermathecal morphology and the fate of stored spermatozoa were studied by microscopic techniques (high-resolution x-ray computed tomography and transmission electron microscopy) in three species with different reproductive modes and lifestyles (*Suocerathrips linguis, Echinothrips americanus, Hercinothrips femoralis*). Mating experiments were conducted to analyse the use of the transferred sperm in the thelytokous *H. femoralis*. Results show that the spermathecae are relatively simple, which can be explained by the availability of sperm and the short lifespan of the females. However, the spermatheca in *H. femoralis* seems to be vestigial compared to the arrhenotokous species and females do not use sperm for fertilisation. No substantial change was observed in the structure of spermatozoa, despite an enlargement of the sperm organelles being measured during storage in all three species. The results of this work demonstrate differences in the morphology of the spermatheca, especially concerning the reproduction mode, promoting the understanding of the complex interaction between morphology and behaviour.

## Introduction

The knowledge about morphology and its implications for behaviour is crucial to understanding the biology of insects. Especially in the case of pest insects, expertise in mating behaviour and reproductive strategy is beneficial for developing new approaches to pest management. Thysanoptera (thrips) is a cosmopolitan order of about 6,300 known species of very small size^1^. It has two suborders, Terebrantia and Tubulifera, and especially the Terebrantia (family Thripidae) has 100 pest species with fast reproduction and rapid generation times. Due to their feeding activity and plant virus transmission, they are causative of billions of dollars of damage^2^. In particular, their cryptic lifestyle and possibility of rapid development of resistance to insecticides make them difficult to control using conventional methods. The Arthropod Pesticide Resistance Database listed resistances in 330 cases within 11 species and against up to 30 different compounds^3^. Thus, the design of new pest-control strategies is necessary, making crucial the understanding of the biology of these insects.

The spermatheca is an adequate target organ for population control because a disruption of the ability to store sperm would lead to a decreased fitness of the female. In general, in insects, the spermatheca (*receptaculum seminis, Samenkapsel*) is mostly a paired or unpaired structure of the ectodermal female gonoduct, which may sometimes have further protrusions^4^. The function of this organ is to store and maintain sperm. Sperm storage enables the female to separate copulation and fertilization concerning time and is a key mechanism underlying the reproductive independence of females from needing to mate several times, the associated costs of mating, and the possibility of post-copulatory sperm competition and cryptic female choice^5^. The extent to which sperm must be kept fertile for the duration of storage should depend on the females’ reproductive strategy. Male ejaculate compounds degrade within a short period and female alone seems to sustain the sperm within the spermatheca^5^. Therefore, species that mate multiple times require low or no capacities and mechanisms to maintain the spermatozoa compared to species with only one or few mating(s) and a long lifespan.

In Thysanoptera, the morphology of the spermatheca is described mainly in species of the suborder Terebrantia^6–11^. The single spherical spermatheca is located dorsally on the vagina. It consists of three different cell types. The pigmented, flat epithelial cells (*Wandzellen*^6^) are responsible for shaping the spermatheca. Cap-like gland cells are mounted on them. Irregular, cylindrical pedestal cells (*Sockelzellen*^6^) form the board seating of the spermatheca, the spermathecal duct and the valve within the duct. The spermathecal duct passes through the pedestal cells. It is strongly compressed at one point by a cuff of resilin with muscle insertions, which presumably function as a pinch cock/squeeze tap (*Quetschhahn*^6^) to release or receive the spermatozoa. In tubuliferan species, the spermathecal complex consists of a large bulb-like spermatheca, connected via a spermathecal duct to the ampoule, and further to the common oviduct^9,12–15^. An additional separate spermatheca-associated gland, as is often found in insects^4^, does not exist in any of the two suborders. The accessory gland of the Terebrantia is an independent structure with a separate duct connecting to the ovipositor, presumably to support egg-laying^6,9,16,17^. After mating, the spermatozoa are bundled in a secretion sheath in the spermatheca of Terebrantia, while they are free in the Tubulifera^5^. Besides Bode’s^18^ first description of the ultrastructure of spermatozoa structure within the spermatheca, nothing is known about what happens to the sperm in the spermatheca within the Thysanoptera during storage.

Species of the suborders of Thysanoptera differ in their life history. Thus, we expect various adaptations of structures and their function concerning reproductive strategy or reproduction mode. Social and subsocial behaviour, like cooperation in carrying the young, overlap of at least two generations and division of labour, is recorded only in Tubulifera^19^. Males and females seem to remate frequently. The remating behaviour is promoted by the morphological adaptation of the synspermatogeny of the males, where sperm is produced during the entire adult lifetime, as well as the large spermatheca of the females^18,20–23^. Additionally, the longer lifespan of some tubuliferan species is likely to require a constant sperm supply over their whole lifetime. Many flower-living Thripidae species (Terebrantia) are more adapted to rapid reproduction and dispersal. Only functional and temporally limited aggregations are known among some Thripidae species, which might aid in finding mates or food sources^2^. Males exhibit prospermatogeny. In this case, the sperm are fully matured with the adult emergence and are therefore only available in limited quantities^18,20–23^. This might constitute an advantage in their short lifespan and the frequently occurred protandry. In addition, in most species, females mate only once or just at very low frequencies^22^ and possess only a small and simply structured spermatheca.

Besides the differences in reproductive strategies, species of the haplodiploid Thysanoptera can also differ in their reproductive mode. Most species reproduce via arrhenotoky, where females arise from diploid eggs, whereas males develop from haploid ones. Some species reproduce by thelytoky, thus females produce only female progeny and no or only very rare males (e.g. *Heliothrips haemorrhoidalis, Hercinothrips bicinctus, H. femoralis, Helionothrips errans, Scirtothrips longipennis, Leucothrips nigripennis, Chaetanaphothrips orchidii*^24^). Some thelytokous or asexual species have a reduced or complete loss of their ability to reproduce sexually. The degree of expression of sperm storage organs in thelytokous, parthenogenetic or non-reproducing insects varies widely. Whereas parthenogenetic reproducing aphids do not possess a *receptaculum seminis* ^25^, some ant species show different degrees of spermatheca reduction^26,27^, non-reproductive females of social wasps and bees and also queens of thelytokous species maintain an intact spermatheca^26,28–32^. Besides structural changes, also the functionality can be affected. Bumblebee workers possess a spermatheca, but lack PAS-positive cells in the spermathecal duct, which might be necessary for the nutrition of the stored spermatozoa^28^. Within the Thysanoptera, there are also great differences. *Heliothrips haemorrhoidalis* have no spermatheca^33,34^. Arrhenotokous males of *Thrips nigropilosus* are not able to copulate with thelytokous females^35^. Antibiotic generated males of the *Wolbachia*-induced thelytokous thrips species *Franklinothrips vespiformis* and *Hercinothrips femoralis* are able to produce and transfer sperm to the female´s spermatheca, but presumably, they are not used for fertilisation^36,37^.

Concerning the differences in reproductive strategies and reproduction modes, an asymmetry in the necessity of maintaining sperm within the spermatheca and their availability should occur and might be reflected in the spermathecal morphology. Thus, in this work, we investigated three species with different reproduction modes and suborder-affiliation (Fig. 5A): *Suocerathrips linguis* (Tubulifera), *Echinothrips americanus* (Terebrantia), and *Hercinothrips femoralis* (Terebrantia). *Suocerathrips linguis* is subsocial with an arrhenotokous reproduction mode. They mate multiple times, show complex behaviours and a relatively long lifespan as subsocial thrips^38–40^. Males produce sperm via synspermatogeny^23,41^, therefore “fresh” spermatozoa should be available over the entire male lifespan. In contrast*, Echinothrips americanus* is a solitary living thrips considered a serious pest due to its wide distribution and large number of host plants^42,43^. Its reproduction mode is also arrhenotokous, but females mate only once and males exhibit prospermatogeny as a possible adaptation to their short lifetime^22,42^. The pest *Hercinothrips femoralis*, also a solitary living insect, is now widespread in tropical and subtropical regions worldwide and common in greenhouses of temperate areas^44^. It reproduces via *Wolbachia*-induced thelytoky^37^, but males can be generated by antibiotic treatment of mothers. We investigated the morphology of the spermatheca of these three species and the structural situation of the spermatozoa within using high-resolution X-ray computed tomography (nano-CT), transmission electron microscopy and light microscopy. We also investigated *H. femoralis* in more detail, to understand to what extent changes due to the thelytoky are visible and whether the sperm of the artificially-generated males are used. Our work enhances the knowledge of these pest insects´ reproduction morphology and contributes in establishing new approaches to their management.

## Materials & methods

### Rearing method

Thrips species were reared at 23 ± 1 °C, 60% relative humidity, light regime L:D 16:8 (light on 6:00 am, 5.000 lux) on species-specific host plants in acrylic cages (50 × 50 × 50 cm, 2 sides covered with fine mesh).

*Echinothrips americanus* was held on potted *Phaseolus vulgaris, Gossypium spp* and *Hibiscus spp*. Commercial organic lemon fruits, *Ocimum basilicum* and *Apium graveolens var. dulce* serve as host plant for *Hercinothrips femoralis. Suocerathrips linguis* was fed on *Sansevieria spp.*

All species were kept in culture at the Martin-Luther University. *Echinothrips americanus* was kindly provided by the University of Wageningen, Netherlands, in 2006. *Hercinothrips femoralis* originate from the nursery of the Pedagogical University Köthen, Germany 1980. To keep the cultures as natural as possible and maintain a degree of variability in the gene pool, animals from other regional populations, such as the botanical garden at the MLU, were regularly added. The colony of *Suocerathrips linguis* was established in 1999 from species found at the Royal Botanical Garden, Kew, London, Great Britain.

### Sample preparation

To obtain mated females with known age, 24 h old unmated females were placed for 48 h with a male in wells of 12-well cell culture plates, each well equipped with a piece of bean leaf and agar. Afterwards, male and female individuals were prepared for analyses.

Development of male *H. femoralis* was artificially induced by antibiotic treatment of their mothers. The detailed protocol and maintaining procedure are described in Krueger & Moritz^23^. Afterwards, F1-Generation of treated mothers was handled as mentioned above.

Because of the subsocial lifestyle of S. *linguis,* it is not possible to rear or mark individuals without massive impact in order to get individuals with known age as in the other two species. Therefore, the colony was regularly checked for mating pairs. Pairs found were picked up with a fine brush and fixed for nano-CT and TEM.

### X-ray micro-computed tomography (nano-CT)

Animals were fixed overnight in cold, fresh 2.5% glutaraldehyde, 2.0% paraformaldehyde, 5% D-glucose in 0.1 M sodium phosphate buffer at pH 7.4. After rinsing the animals three times in buffer solution for 5 min, postfixation in 1% OsO_4_ solution (same buffer) was conducted chilled on ice for 4 h followed by dehydration in a graded series of ethanol. After washing in absolute ethanol, samples were critical point dried (Emitech K 850, Quorum Technologies, United Kingdom) and subsequently mounted on insect pins. High-resolution Zernike phase-contrast X-ray computed tomography (nano-CT) imaging was performed on a Carl Zeiss Xradia 810 Ultra equipped with a 5.4 keV chromium X-ray source using a field of view of 64 × 64 µm^2^ for all samples, with an isometric voxel size of 64 nm (no binning), 901 or more projections and 30 s projection exposure time for *E. americanus*, and isometric voxel size of 128 nm (binning 2), and 15 s projection exposure time for *H. femoralis* and *S. linguis*. The image reconstruction was performed using the filtered back-projection algorithm in the XMReconstructor software integrated to the device, and the tomograms obtained were exported as a stack of 16-bit TIFF images. The commercial software Avizo (Thermo Fischer Scientific, version 9.4.0) was used for image correction, segmentation, 3D rendering and for preparing the videos (Supplementary material). Image correction was performed using non-local means filter. Image segmentation was performed using a combination of contrast-based voxel selection and contrast-based user-selection propagation (magic wand and watershed tools of Avizo).

### Light- and transmission electron microscopy

Abdomens were cut-off with a razor blade and fixed in 2.5% glutaraldehyde, 3% glucose buffered in 0.1 M cacodylate overnight at 4 °C. After postfixation in 2% OsO_4_, samples were dehydrated and embedded in Epon 812 resin via acetone.

Sections were made with an ultracut microtome (UltracutR, Leica, Germany). Semithin-sections were stained with toluidine blue, and ultrathin-sections with uranyl acetate. A Leitz DMBRE (Leica, Germany) fitted with a DFC 450C camera (Leica, Germany) and a Jeol TEM 1010 (at 80 kV) equipped with a Megaview III camera were used for observations.

### Comparison of ultrastructure spermatozoa in males and females

Length and width of the conspicuous organelles (acrosome, nucleus, mitochondria, doublets of the axonema) of the spermatozoa were measured from TEM-images. Due to the different spermiogenesis of the species (see Krueger & Moritz 2021) and the comparability, only measurements from the male adult testes (*E. americanus* & *H. femoralis*) or the seminal vesicle (*S. linguis*) were used for evaluation.

The size of the organelles within the spermatozoa varies with their position. To compare the size of spermatozoa organelles between males and females, regions of interest were defined (see Fig. 5A, C). Only morphologically intact spermatozoa were used for measurements, which were defined as spermatozoa with distinct outer membrane boundaries and typical compact organ structure (see Krueger & Moritz^23^). Measurements were done with Fiji-Software^45^. Since the changes in organelle length and width were shown to be relative, the calculated areas are shown in µm^2^ (length x width / 1,000,000) for a clearer representation in the figures.

### Sperm functionality in H. femoralis

To test the functionality of the sperm of artificially generated *H. femoralis* males, cross-mating experiments were conducted (Fig. 6A).

The following conditions were analysed:

1. antibiotic-generated female + antibiotic-generated male (F-AB + M-AB)
2. unmated antibiotic-generated females (F-AB)
3. female + antibiotic-generated male (F + M-AB)
4. unmated female (F)

Antibiotic-generated individuals were obtained by feeding the F0-generation mothers (n = 40) with rifampicin as mentioned in Krueger &Moritz^23^. The F1 generation was raised individually in formerly mentioned cell culture multiwell plates and sexed after adult emergence. Untreated individuals were obtained by separating larvae from the main culture and raising them under the same conditions as the treated ones.

Two to five days-old males and females (F1) were mated or remained unmated according to the scheme in Fig. 6A (15 individuals per group). Pairs were brought together in fresh prepared multiwell plates and observed until successful copulation. Successful copulation was considered as visible inserted aedeagus lasting more than 5 min. Males were removed 24 h after initial copulation. Females were allowed to lay eggs for consecutive 5 days. Afterwards hatched larvae (F2-generation) of each female were counted every two days and transferred in PP-specimen cups. Specimen cups (volume 100 mL) were filled with 20 mL of 1.4% agar (*w/v*). Agar surface was covered with parafilm to prevent drowning of larvae. A primary leaf of *Phaseolus vulgaris* served as a food source and was pricked into the agar with the petiole. Larvae were raised until adult emergence in climate chambers (23 ± 1 °C, 50% RH, light regime L:D 16:8, 5.000 lx). Non-hatched eggs were counted 10 days after the initial egg-laying period by heating the leaf discs floating in water in a household microwave until the leaves became translucent. Non-hatched eggs occur as white spots under a binocular. Male–female sex ratio and total fecundity (hatched larvae + non-hatched eggs) were recorded for each female. Females without progeny were discarded from further analysis.

### Statistics

The comparison of the ultrastructure of spermatozoa obtained from the testis/seminal vesicle and the spermatheca was performed with non-parametric tests (Mann–Whitney-U-test, Kruskal–Wallis-test, p < 0.05), because of non-normal data distribution (Shapiro–Wilk-test, p < 0.05). Also, the fecundity and male sex ratio of *H. femoralis* progeny were analysed with Kruskal–Wallis-test (p < 0.05, but for pairwise comparison of mating groups, p was Bonferroni-corrected).

## Results

### Structure of Spermatheca

**Figure 1:**
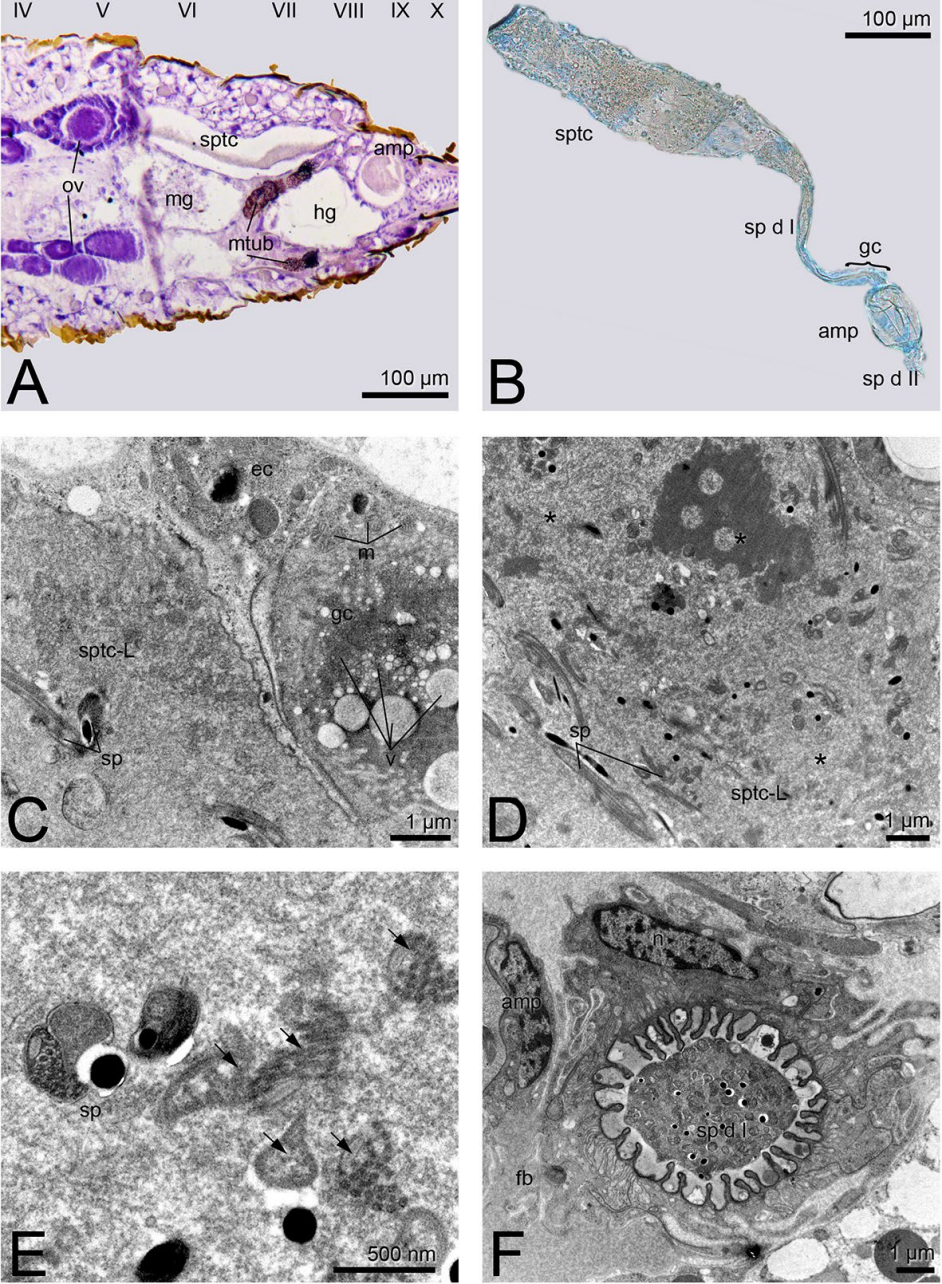
*Suocerathrips linguis*, (**A)** position of spermatheca and ampoule in the female’s abdomen, frontal section of segments IV to X; (**B)** dissected spermatheca and ampoule with connecting ducts; (**C**) ultrastructure of spermatheca in the border area between lumen, gland- and epithelial cell, (**D**) lumen of the spermatheca filled with secretion and spermatozoa; (**E**) close up of intact and defective sperm within the spermatheca lumen; (**F**) spermathecal duct which connects the spermatheca and the ampoule filled with secretion and sperm, transverse section.

**Figure 2:**
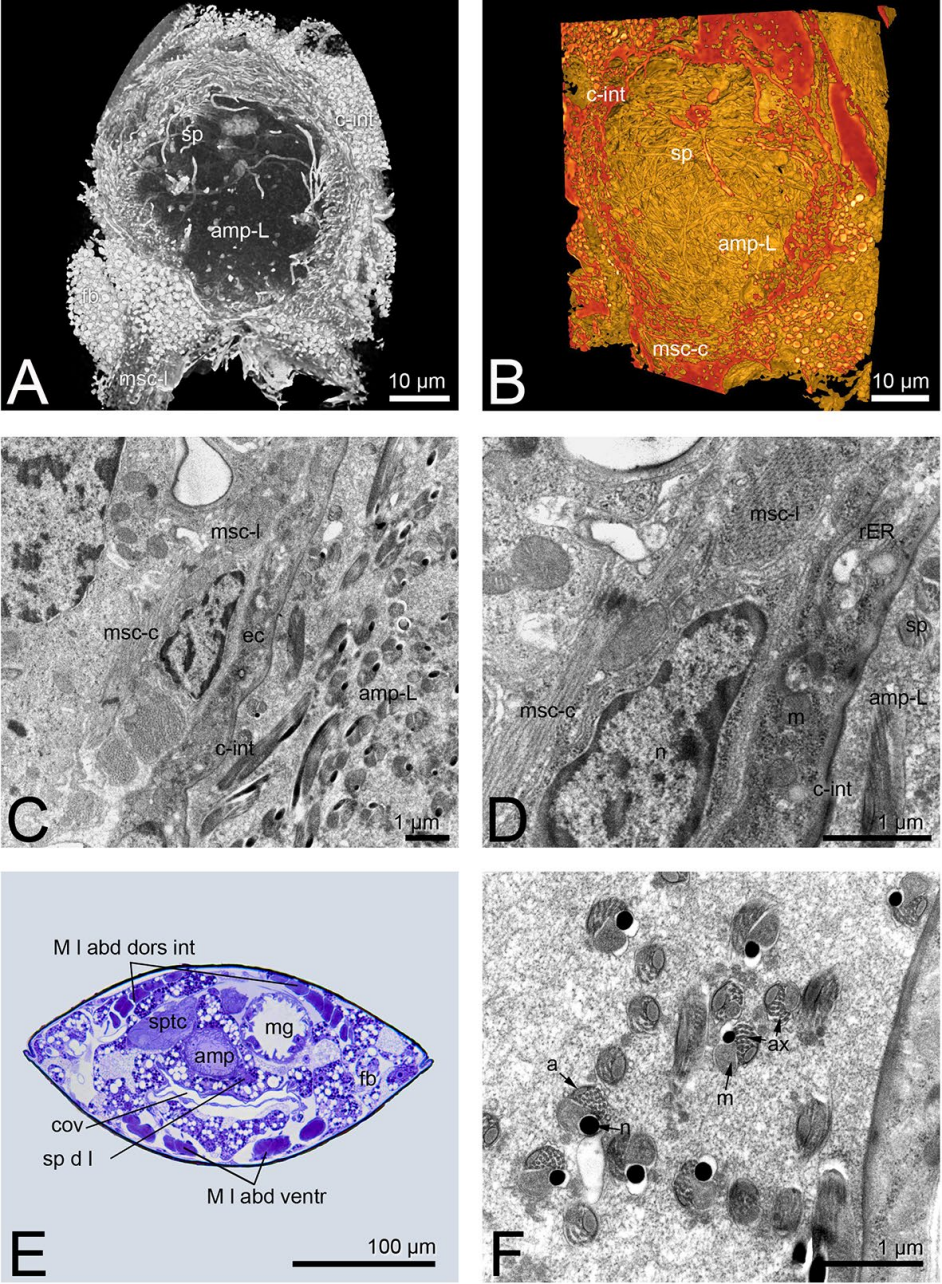
*Suocerathrips linguis*, (**A**) ampoule, nano-CT frontal section; (**B**) ampoule filled with sperm, nano-CT oblique view; (**C**) border area of the ampoule with the lumen and the three-layered epithelial structure, (**D**) epithelial layer consisting of epithelial cells, muscle cells and a connection layer to the fat body; (**E**) location relationship of ampoule and spermatheca within the body cavity, transverse section; (**F**): close up of spermatozoa ultrastructure within the ampoule.

The spermathecal complex in the *Suocerathrips linguis* consists of two parts connected by a duct (Fig. 1A, B). The proximal part (hereafter referred to as spermatheca) is an elongated blind-ending tube (400 µm × 85 µm) located dorsal left in the abdominal segments VI to VIII (Figs. 1A, B; 2E). The epithelial layer is unicellular with single intercalated glandular cells. While the wall-building cells are characterized by ER, mitochondria, and glycogen, the gland cells have large amounts of secretions and vesicles, especially fat inclusions (Fig. 1C), which can already be seen in the fresh preparation (Fig. 1B). Spermatozoa are embedded in large amounts of secretions, which appear in different structures and densities under the electron microscope (Fig. 1C–E). In addition, defective spermatozoa (recognizable by the deformation of the spermatozoa and the dissolved membrane boundary) occur in large numbers, especially in the proximal section (Fig. 1D, E).

Distally a 200 µm long duct (here spermathecal duct I) is adjoined by an ellipsoid, here called ampoule. The duct is formed in the proximal region by epithelial cells, whereas glandular cells are found in the 90 µm long distal region, located directly at the entrance to the ampoule (Fig. 1B, F). The glandular part of the duct is characterized by cuticular and membrane infoldings, as well as large amounts of mitochondria, ER and glycogen. Spermatozoa and secretions seen with darker grey tones, due to the staining with OsO_4_, are observed within the lumen (Fig. 1F).

The ampoule is about 85 × 60 µm in size and located centrally in the abdominal segments VIII to IX (Figs. 1A, 2A-D). The outer epithelium consists of three layers (Fig. 2C, D). The ampoule-lumen is delineated by a cuticular intima, followed by an epithelial monolayer characterized by rough endoplasmic reticulum, mitochondria and glycogen granules. Sitting on these, a muscle layer with longitudinal and circular fibres is present. A third cell layer forms the completion and the connection to the fat body. It is characterized by fat inclusions and other vesicles (Fig. 2A). The lumen of the ampoule is filled with secretion, whereas spermatozoa are located in the peripheral area (Fig. 2A,B, Supplementary Video 1). The spermatozoa lie here without any recognizable order (Fig. 2A,B, Supplementary Video 1).

**Figure 3:**
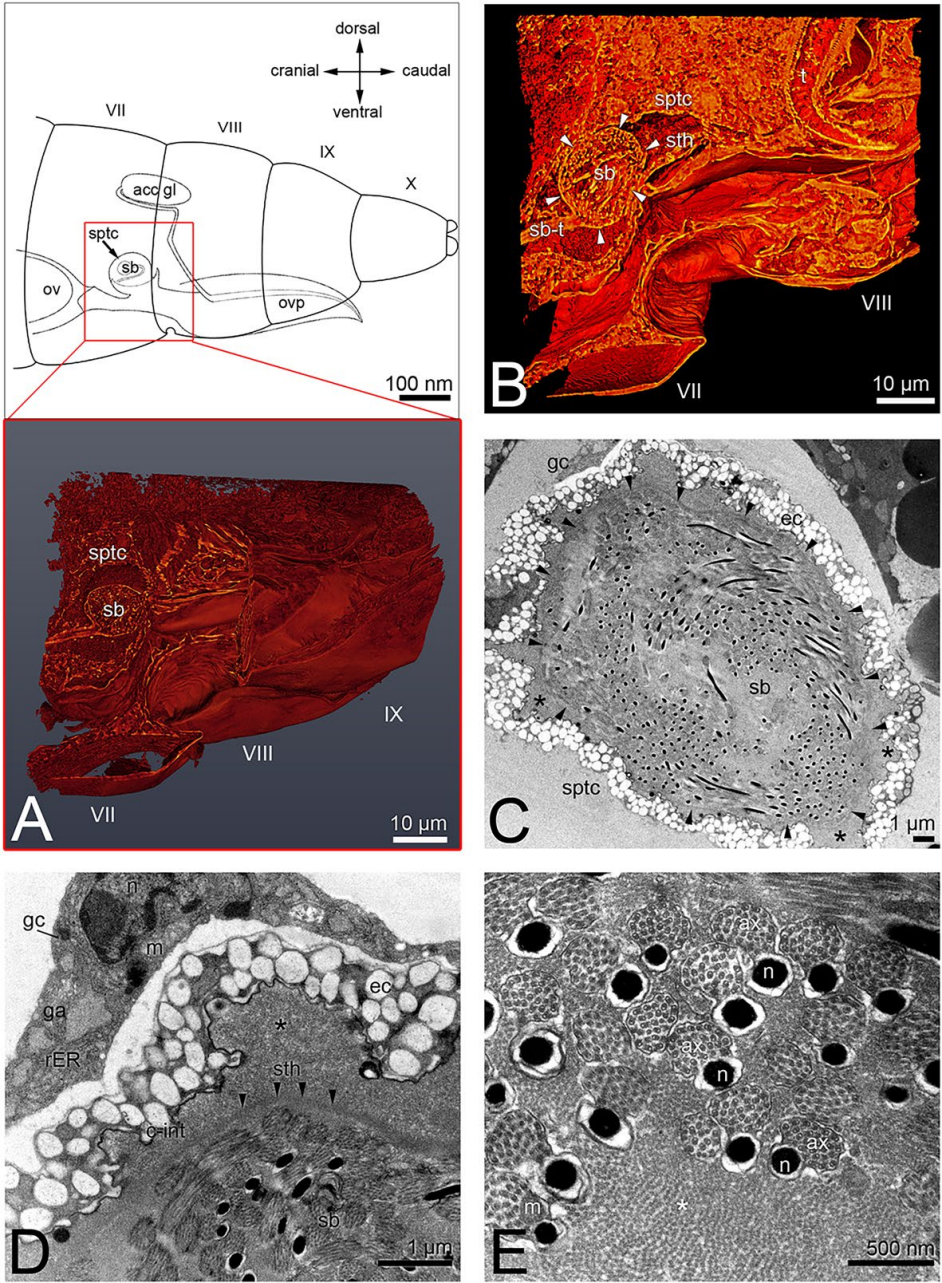
*Echinothrips americanus*, (**A**) schematic drawing of the spermathecas position within the segment VII and VIII, A inlay: nano-CT image of the region VII to IX with spermatheca and sperm ball inside, (**B**) compact sperm ball made of compressed secretion within the spermatheca, nano-CT; (**C**) spermatheca with sperm ball inside (arrowheads) and secretion (*), (**D**) spermatheca epithelial layer with pigment granules and gland cells, (**E**) spermatozoa within the sperm ball.

In *Echinothrips americanus,* the spermatheca is roughly spherical with a size of 25 × 20 µm, depending on mating status (Fig. 3A–C). It is sitting on the vagina in segment VII/VIII and is connected by a duct (Fig. 3A,B). The outer wall is built by a single layer of epithelial cells, characterized by pigment granules and inwardly completed by a chitinized intima (Fig. 3C,D). The pigment granules also account for the brownish colouration of the spermatheca seen in fresh preparations. Besides the nuclei and some mitochondria, no other organelles are obvious in this cell type. Single gland cells are found on the outside of the epithelial layer (Fig. 3C,D). Large amounts of rER, mitochondria, vesicles and some Golgi apparatus are noticeable (Fig. 3D). In mated females, the spermatheca is filled with darker stained secretion (Fig. 3C,D). Sperm enclosed in a secretion mass (hereafter sperm ball) is visible (Fig. 3; Supplementary Video 2).

The sperm ball is delimited from the outside by secretion compression (Fig. 3B–D). Within the sperm ball, the spermatozoa are arranged circularly in a granular appearing secretion (Fig. 3D,E; Supplementary Video 2).

**Figure 4:**
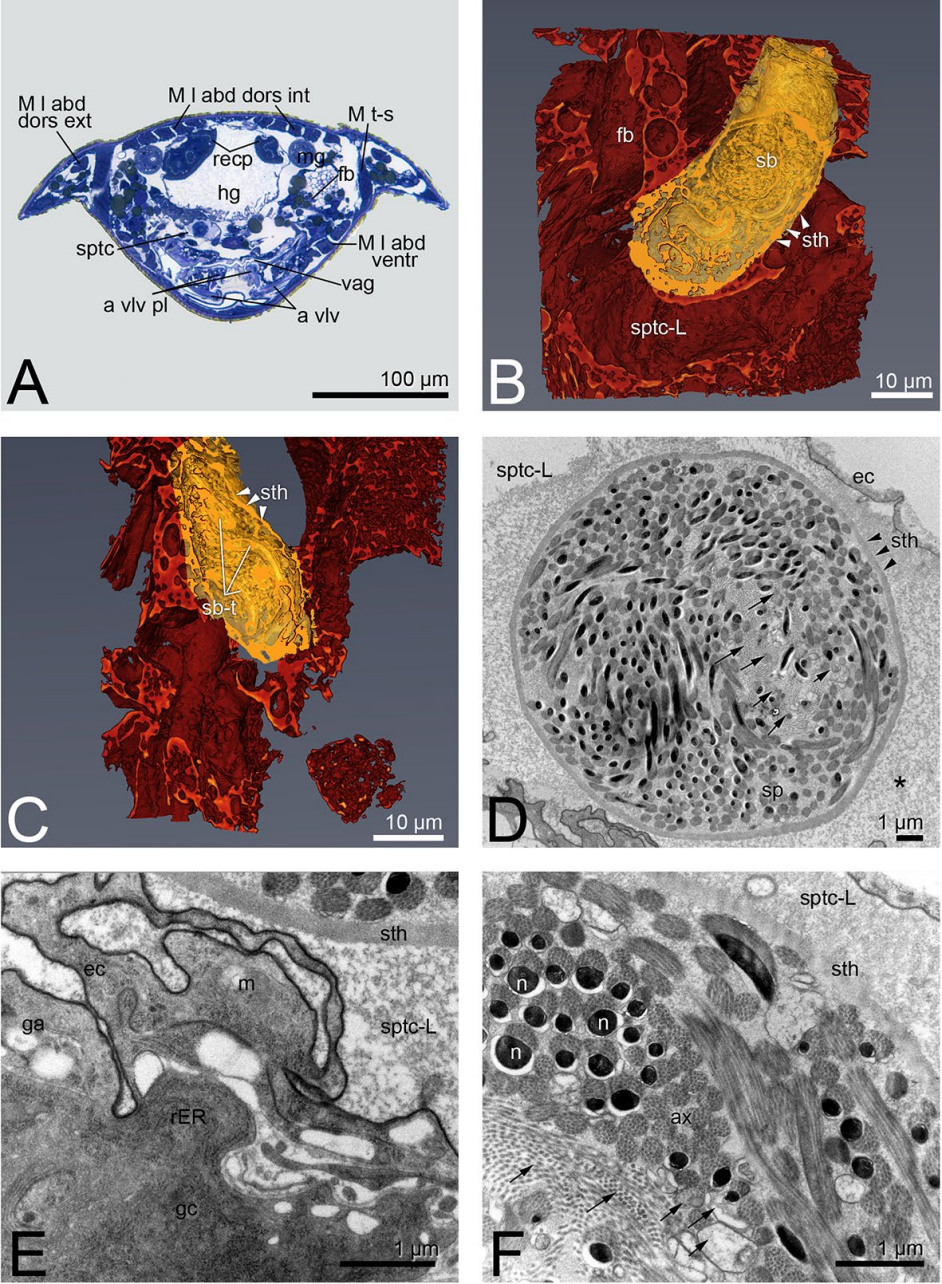
*Hercinothrips femoralis*, (**A**) location relationship of the spermatheca within the body cavity, transverse section; (**B**) spermatheca filled with a sperm ball of compressed secretion and spermatozoa, nano CT; (**C**) spermatheca filled with a sperm ball with several tubes; (**D**) sperm ball within the secretion-filled spermatheca; (**E**): epithelial layer of the spermatheca; (**F**) ultrastructure of defective and intact spermatozoa within the sperm ball.

In *Hercinothrips femoralis,* the ellipsoid spermatheca (65 × 25 µm) is sitting on the vagina in segment VII/ VIII. The structure is similar to that of *E. americanus*. The spermatheca is constituted by a layer of thin epithelial cells (Fig. 4B,C), topped with single gland cells (Fig. 4E). Mitochondria, large amounts of rER and Golgi apparatus are obvious within the gland cells. In the mated females, the spermatheca lumen is filled with light and homogenous secretion (Fig. 4D,E) and a sperm ball (Fig. 4A–D, Supplementary Video 3). The sperm ball is encased by a dense secretion layer. Spermatozoa are compactly packed inside in a circular arrangement. The secretion inside is less electron-dense structured than in *E. americanus* (Fig. 3E). Several sperm ball tubes can be seen within the spermatheca (Fig. 4C; Supplementary Video 3).

### Comparison of sperm derived from males and females after mating

**Figure 5:**
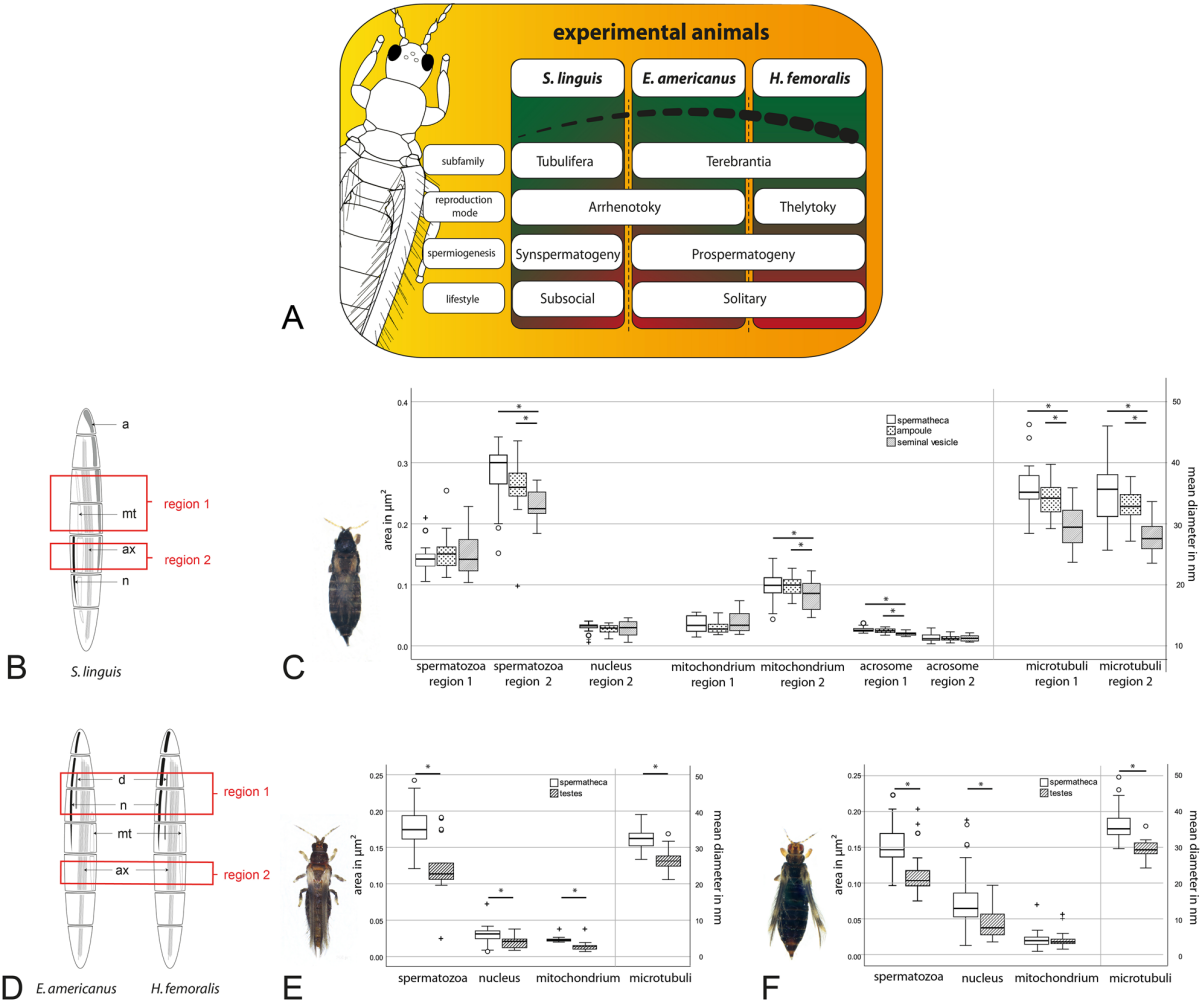
Comparison of the ultrastructure of the spermatozoa in the testes/seminal vesicle and the spermatheca/ampoule. (**A**) overview of the experimental animals and characteristics; (**B**,**D**) Overview of the sperm ultrastructure of the three species from anterior to posterior. by Krueger & Moritz 2021, used under CC by 4.0 /Distance, labelling and marking changed. The regions marked were selected for detailed measurements and comparison between the structure of spermatozoa obtained from males and females; (**C**) *Suocerathrips linguis*, measurements of the organelles within the different regions and origins, (n (sptc) = 34, n(amp) = 52, n(seminal vesicle) = 28); (**E**): *Echinothrips americanus*, (n(sptc) = 98, n(testes) = 24); (**F**) *Hercinothrips femoralis*, (n(sptc) = 79, n(testes) = 17); * indicate significant differences, (A: Kruskal–Wallis -test, p ≤ 0.05, B/C: Mann–Whitney U-test, p ≤ 0.05), ○ and + indicate (extreme) outliners.

We defined regions of interest within the spermatozoa itself to compare the morphology of the spermatozoa enclosed by the female spermatheca and the male testes resp. seminal vesicle. Within the two defined regions, the sperm organelles have their maximum size^23^(Fig. 5B, D).

In males` testes, the threadlike spermatozoa bear helicoidally arranged organelles, consisting of a nucleus, mitochondria, axoneme and, in Terebrantia, an electron-dense body, and in Tubulifera an acrosome. The unusual axoneme structure is most striking: 27 microtubules, built from 9 doublets with dynein arms, 9 doublets without and 9 singlets. Microtubule elements are arranged in an asymmetric pattern, beginning with a single microtubular singlet and ending with an arm-less doublet.

In *Suocerathrips linguis*, spermatozoa within the males´ seminal vesicle and females’ spermatheca complex bear the same organelles (Fig. 2F). The structures mainly retain their integrity even in the spermathecal complex. Only in the spermatheca single defective spermatozoa are observable. Within the spermathecal complex, the transferred spermatozoa increase in size in region 2 compared to the situation in the males´ seminal vesicle. Also, the size of the mitochondrion and acrosome increase, but only in one of the two regions. In contrast, the diameter of the microtubules significantly increases in both regions (Fig. 5C).

As in *S. linguis*, the ultrastructural organization of the spermatozoa after mating did not change in *E. americanus* and *H. femoralis*, but the size of the spermatozoa and the organelles differed significantly (Fig. 3E, 4F). All organelles, except the mitochondrion in *H. femoralis*, increase in size after the transfer of the spermatozoa to the spermatheca (Fig. 5E, F). However, several defective spermatozoa are observable within the sperm ball in *H. femoralis* (Fig. 4F).

### Sperm functionality in H. femoralis

The males of *Hercinothrips femoralis* were artificially generated by antibiotic treatment of the mothers to push back the thelytoky-inducing Wolbachia bacteria. It would clarify whether the spermatozoa are capable of fertilisation since defects are already evident in the testes^23^ and also in the spermatheca (Fig. 4F). The fertility and sex ratio of the offspring in different cross-mating designs were investigated.

**Figure 6:**
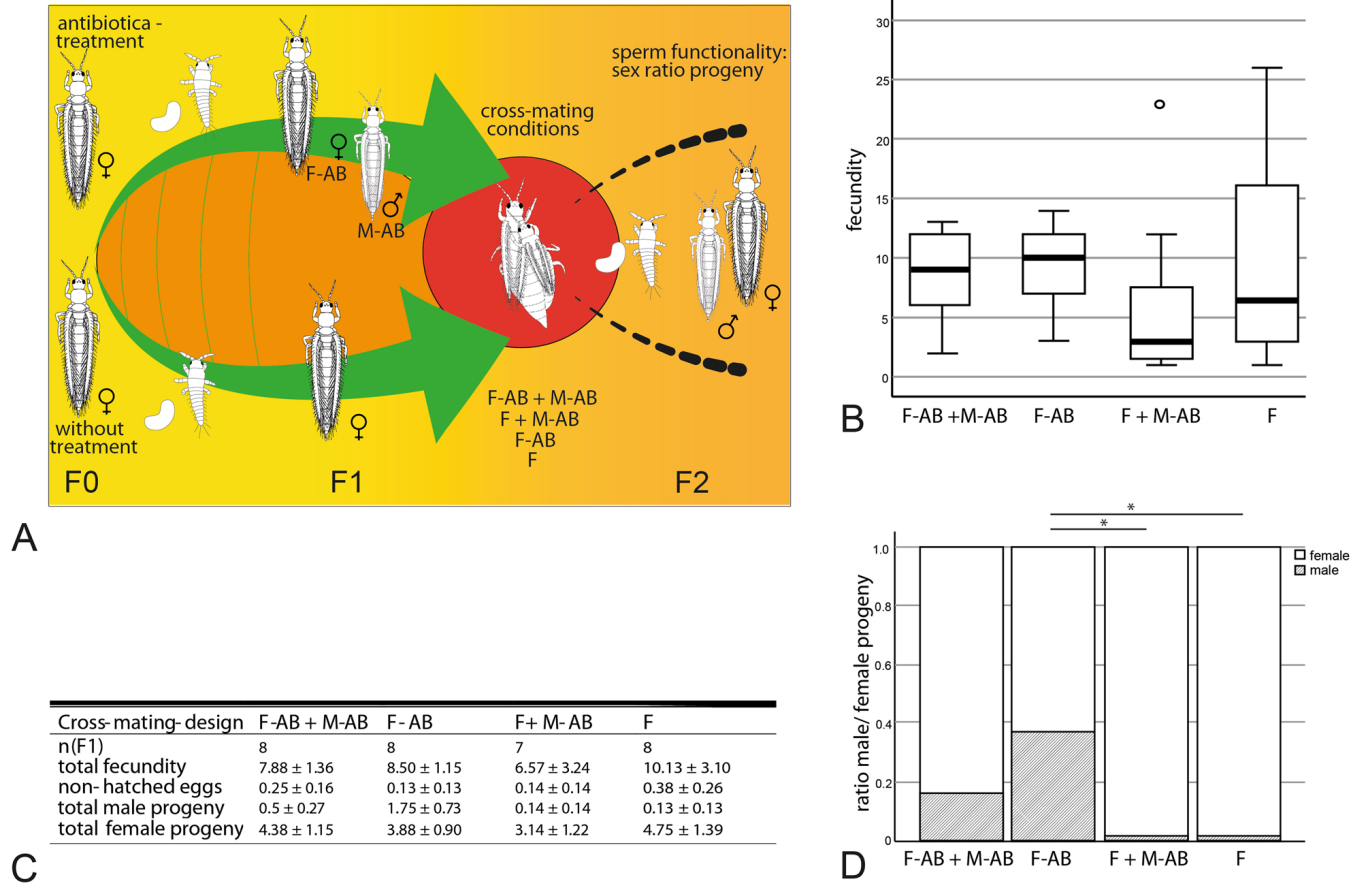
sperm functionality in *H. femoralis*, (**A**) Scheme of the experimental setup in *H. femoralis*: generating males and females (F1) by antibiotic treatment of mothers (F0), cross mating conditions and recording of the sex ratio in the F2 generation for the analysis of sperm functionality; (**B**) fecundity (F1) depending on cross-mating design group; (**C**) data of experiment sperm functionality depending on females cross-mating design group; (**D**) male and female sex ratio of F2, depending on mothers cross-mating design group.

The female total fecundity did not differ significantly between mating designs (Kruskal–Wallis-test, H = 1.91, p = 0.59) (Fig. 6B,C), as well as the number of non-hatched eggs (Fig. 6C). In contrast, the male sex ratio of the progeny differs significantly between the groups (Kruskal–Wallis-test, n = 31, H = 10.692, p = 0.014). The posthoc test (Dunn-Bonferroni-test) reveals the differences between the groups F & F-AB (H = 2.863; p = 0.025), as well as F + M-AB & F-AB (H = 2.764; p = 0.034) (Fig. 6D).

## Discussion

In this work, we investigated the morphology of the spermatheca of three species (in two suborders) of thrips (Fig. 5A): *Suocerathrips linguis* (Tubulifera), *Echinothrips americanus* (Terebrantia), *and Hercinothrips femoralis* (Terebrantia). The examined representatives of the two suborders show the typical structure of the spermathecae in each respective case and differ very clearly.

So far, the spermathecal structures in the Tubulifera are poorly described. The two-part spermathecal complex consists of the spermatheca and an ampoule, which are connected by a duct. The exit of the complex opens directly at the end of the common oviduct since Tubulifera does not have a vagina in the narrower sense^9,12,46,47^. The size of the spermatheca in *Suocerathrips linguis* is in the intermediate range compared to other tubuliferan species, but is very large compared to the body size (Table 1). The morphology resembles that in Tubulifera, best described in *H. verbasci* with the left-side location in abdominal segments VI to VII, the cellular structure and the loosely arranged spermatozoa inside the lumen^9^. Like the spermatheca, the ampoule of *S. linguis* is of intermediate size, but large when compared to the body size in (85 × 60 µm), compared to other species described (Table 1). It contains spermatozoa, but no spermatheca-like secretion^7^. The detailed function of this structure is unknown, but the ring muscles of the ampoule and a presumed pumping function were already noticed by Jordan^12^. De Marzo also attributes this function to the structure by naming it a “pumping device”^47^.

**Table 1:** Overview of previously described spermatheca sizes and shapes in Thysanoptera.

Suborder	Family	Species	Shape*^1^	Spermatheca size in µm	Ampoule size in µm	Method*^2^	*^3^	Body size in mm	*^4^	Body size/max. spermatheca relation in %
Tubulifera	Phlaeothripidae	*Bactrothrips buffai*		330 × 77–132	160 × 23–33	d	^13^	4.5	^13^	Spermatheca: 7.3
										Ampoule: 3.6
		*Compsothrips albosignatus*	a	750 × 350	100 × 80	e	^47^	4.3–4.5	#	Spermatheca: 16.6–17.4
										Ampoule: 2.2–2.3
		*Haplothrips simplex*	a	595 × 70–110	20 × 30	e	^47^	3	^51^	Spermatheca: 19.8
										Ampoule: 1.0
		*Haplothrips verbasci*	a	160 × 75–105	10–20 × 11–17	d	^9^	1.80–1.90	^52^	Spermatheca: 8.4–8.9
										Ampoule: 1.05–1.1
		***Suocerathrips linguis***	**a**	**400 × 85**	**85 × 60**	**e**		**1.8**	^53^	**Spermatheca: 22.2**
										**Ampoule:4.7**
Terebrantia	Aeolothripidae	*Aeolothrips cursor*	c	54 × 58	-	e	^47^	0.91–1.07	^54^	5.4–6.4
		*Aeolothrips intermedius*	b	100 × 100 × 100	-	d	^7^	1.85–2.4	^54^	4.1–5.4
		*Aeolothrips tenuicornis*	c	130 × 140	-	e	^47^	1.92–2.52	^54^	5.6–7.6
	Melanthripidae	*Melanthrips fuscus*		50 × 50	-	e	^47^	1.8–2.23	^54^	2.2–2.6
		*Ankothrips mavromoustakisi*		21 × 21	-	e	^47^	1.68–1.94	^54^	1.1–1.3
	Thripidae	Subfamily: Thripinae								
		***Echinothrips americanus***	**c**	**25 × 20**	**-**	**d**		**1.36–1.6**	^54^	**1.6–1.8**
		*Frankliniella fusca*	c	28–50 × 28–50	-	d	^9^	1.25–1.53	^54^	3.3–4.0
		*Frankliniella occidentalis*	c	27–28 × 27–28	-	e	^11^	1.44–1.82	^54^	1.5–1.9
		*Limothrips cerealium*	c	42 × 42	-	f	^55^	1.68–2.23	^54^	1.9–2.5
		*Neohydatothrips gracilicornis*	c	16 × 18	-	e	^47^	1.38–1.66	^54^	1.1–1.3
		*Pezothrips kellyanus*	c	45 × 50	-	e	^47^	1.63–2.18	^54^	2.3–3.1
		*Thrips physapus*	c	40 × 40	-	d		1.25–1.7	^54^	2.4–3.2
		Subfamily: Panchaetothripinae								
		***Hercinothrips femoralis***	**c**	**65 × 25**	**-**	**d**		**1.22–1.63**	^54^	**4.0–5.3**

Species examined in this study are indicated in bold.

*^1^Shapes: a: two-parted with spermatheca and ampoule, b: rocking-horseshoe shaped spermatheca, c: round to pear-shaped spermatheca.

*^2^Method of spermatheca measurement: d: chemical fixation, e: fresh preparation, f: unknown.

*^3^Reference of spermatheca measurement.

*^4^Reference of body size (female), all measurement come from total preparations.

#Own measurement from samples of G. Moritz collection

Body size/ max. spermatheca relation in % was calculated by using the maximal length of the spermatheca and the minimum and maximum range of the body size as a proxy for the relative size of the spermatheca.

In contrast, the spermathecal complex of the tubuliferan species *Bactrothrips buffai* (formerly named *Caudothrips buffai*) differs from the other described species. The bulblike spermathecae merge into a vesicular dilated structure, which exit in a long duct, lined with cuticular ring clasps. The duct ends with a cuticular valve-process which provides a long connection to the common oviduct^13^. Organs have been assigned the wrong function in some publications, maybe due to the different structure to the better studied Terebrantia. Melis considered in *Liothrips oleae* the spermathecae as a spermathecal gland^48^, whereas Gehlsen referred to the spermatheca as mycetoma in *S. linguis*^40^.

In Terebrantia, especially in the Thripidae, the spermatheca consists of a single spherical structure connected via a spermathecal duct to the vagina on which it sits socket-like^6,9,11^. The Aeolothripidae species *Aeolothrips intermedius* possesses a rocking-horseshoe shaped spermatheca^7^, but the morphological structure seems to be similar to the Thripidae situation. *Echinothrips americanus* fits into the described scale of thripid species, whereas the lumen of the spermatheca in *H. femoralis* is bigger (Table 1). In this work, the typical cellular structure of the spermatheca consisting of three cell types^6,11^ was visible in *Echinothrips americanus.* Also, the pigment granules were evident, which are causal for the orange-brown colour in fresh preparations of the terebrantian spermatheca^6,9,11,12^. In contrast, the epithelial cells in *H. femoralis*, which are forming the wall of the spermatheca, are not so well developed or smaller. Also, the proportion of pigment granules was lower. This less prominent construction, compared to other terebrantian species, might be causative in the thelytokous reproduction mode. Previously, effects of thelytoky regarding the spermathecal morphology in thrips were only assigned to the size of the spermatheca. In the thelytokous *Parthenothrips dracaenae* the small spermatheca size was attributed to the reproduction mode^41^, as well as in *H. femoralis*^8^. In both cases the spermatheca was measured unfilled and it´s known from Bode, that the terebrantian spermatheca hardly has any lumen in the unmated state^6^.

Despite the asexual reproduction of *H. femoralis*, they maintain their storage organ, similarly to several asexual species^49^. Due to relaxed selection pressure and pleiotropy or drift, it seems to have only low costs to maintain these organs even under thelytoky^49,50^.

The different storage mechanisms of spermatozoa in the spermatheca, which are loosely arranged in tubuliferans and packed in a dense secretion layer in terebrantian species^6,9–11^, were evident in the species studied here. In Terebrantia, the secretion is derived from the male, which coagulates in the female’s spermatheca, and adopts the shape of a ball^22^. It thus corresponds to a so-called “female-determined type 1 spermatophore” according to Gerber^22,56^. However, the thelytokous *H. femoralis* shows an altered shape of the sperm ball with several exit ducts.

From the ultrastructure of the most spermatozoa within the spermathecae, it can be seen that all organelles are present as in the male^18,23,57–60^. However, defective sperm was observable in the proximal region of the spermatheca of *S. linguis* and the whole area of the sperm ball in *H. femoralis.* As male *S. linguis* showed an intact spermiogenesis, the defective sperm is not received from the male. It might be “old” sperm, which has not been used for fertilization and is degraded over time, maybe because of the structure of the spermatheca as a long blind sac. In contrast, in testes of male *H. femoralis* defective spermatozoa in a similar amount can be seen and might be transferred to the female spermatheca with mating.

Our study shows for the first time a measure of the sperm ultrastructure within the spermatheca of the Thysanoptera to analyse possible changes. The spermatozoa within the spermatheca increased in size as a whole, as well as in the individual organelles in all three species, irrespective of the reproduction mode. The non-significant change in various regions in *S. linguis* may be due to the larger sperm size and the different organelle arrangement compared to the terebrantian species. The helical arrangement of the organelles and the presence of the acrosome can lead to the displacement of the organelles during the swelling process. However, the conspicuous swelling of the spermatozoa and organelles may represent a post-ejaculatory modification to sperm (PEMS)^61^. PEMS is defined as biochemical, physiological and/or structural modifications to sperm after ejaculation, but excludes modifications to sperm that are attributable to sperm-egg interaction^61,62^. Thus, morphological changes of the spermatozoa in the female are also found in insects, like cockroaches, grasshoppers, flies and butterflies^63–66^. Even though the morphological changes are described here as a capacitation-like process, the term should only be applied in a narrower sense to mammals^61^. Often the axoneme and motility are affected by PEMS, which leads to a better motility^63,65–68^. This apparent necessary PEMS response in the female may be the cause of the frequent finding of low motile or immotile spermatozoa in fresh preparations of male Thysanoptera testes. Previously this observed low motility was attributed to the unusual 9 × 2 + 2 axoneme arrangement^10,18^.

Besides the structural changes in the spermatheca, the functionality of the sperm was tested in *H. femoralis.* In our study, the male sex ratio of progeny in antibiotic-treated females (F-AB) increases, as suggested under Wolbachia-induced thelytoky and also shown in Kumm & Moritz^37^. Mating of thelytokous untreated females with antibiotic-generated males leads to almost exclusively female offspring, as in unmated thelytokous females (F + M-AB & F). This phenomenon is also described in *Leptopilina clavipes* (Hymenoptera: Figitidae) and *Telenomus nawai* (Hymenoptera: Scelionidae)^69,70^.

The disuse of spermatozoa or failure in fertilization could be driven either by the male or the female. In the case of the male, in *Hercinothrips femoralis* spermiogenesis seems to be partially defective, while intact mature spermatozoa occur with defective ones in the male testes of antibiotic-generated males^23^. Producing immotile spermatozoa or having dysfunctional spermiogenesis are typical traits under parthenogenesis^49^.

In the case of the females, it is presumed that asexual females have lost their ability to fertilise their eggs regarding loss of necessary alleles or change in egg morphology ^49,71^. The asexual bushcricket *Saga pedo* females produces eggs with a reduced number of micropyles compared to sexual ones, which limits the chance of egg-sperm interactions^72^. Other species lack the possibility to maintain sperm within the spermatheca^28,73^. The cryptic female choice or population incompatibilities can also be reasons for the disuse of the transferred spermatozoa^49^. In *Timema* stick insects, sperm from asexually produced males in crosses with sexual females show a reduced efficiency compared to crosses with sexual females^75^. A connection to cryptic female choice or population incompatibilities is discussed, but both mechanisms can´t be individually evaluated^49,75^. Female terebrantian Thysanoptera possesses a muscle insertion at the spermathecal entrance, which may enable them to control the decision about fertilization^6^. Cryptic female choice by sperm exclusion would therefore be conceivable. However, looking at the slight (although not significant) higher male sex ratio in mated-antibiotic treated females (F-AB + M-AB & F + M-AB), compatibility issues caused by the *Wolbachia* infection may also be the cause. Parthenogenesis inducing Wolbachia infection can be polymorphic, whereas in some species the chromosomal effects are supressed when (antibiotic-generated-male) sperm fertilize eggs, but in other species, the infection is fixed within the species, which results in parthenogenesis independent of sperm presence^36,74,76^. The exact chromosomal mechanisms for diploidisation of eggs under *Wolbachia* influence in thrips are still unknown and require further study.

In conclusion, the species studied here show the suborder-typical spermathecal morphology, but the presumed asymmetry in maintaining the sperm and their reflection in the morphology is complex, and long-term storage of sperm appears to play a minor role. *S. linguis* possess a large spermathecal complex though it is equipped only with a few glandular cells. Thus, effective sperm maintenance seems not to happen, and defective spermatozoa are located especially in the proximal region. As a sub-social species, they live in groups, have a relatively long lifespan for thrips and mate multiple times. It does not seem necessary to store sperm effectively because of the constant availability of males and the multiple matings. As both terebrantian species represent pest insects with rapid reproduction and short generation times, the spermatheca has a simple structure with only a few glandular cells for producing substances to maintain sperm. In addition, *H. femoralis* possess an underdeveloped spermatheca due to their thelytokous reproduction mode. However, intact sperm seems to be stored normally in *H. femoralis* with a comparable PEMS reaction, as in the arrhenotokous species *E. americanus*. Further studies are needed to investigate whether the demonstrated PEMS reaction revealed here for the first time in Thysanoptera could be a new target for management strategies. If the reaction is prevented, activation and thus fertilization might fail to occur, which would reduce female fitness and change the population dynamics.

## Supplementary Information

Supplementary Information 1.

Supplementary Video 1.

Supplementary Video 2.

Supplementary Video 3.

## Data Availability

The data presented in this study are available upon request from the corresponding author.

## Abbreviations

a: Acrosome
acc gl: Accessory gland
a vlv: Anterior ovipositor valve
a vlv pl: Dorsal process of anterior ovipositor valve
amp: Ampoule
amp-L: Ampoule lumen
ax: Axoneme
c-int: Cuticular intima
cov: Common oviduct
ec: Epithelial cell
fb: Fat body
ga: Golgi apparatus
gc: Gland cell
hg: Hindgut
m: Mitochondrion
M l abd dors int: Musculus longitudinalis abdominis dorsalis interior
M l abd dors ext: Musculus longitudinalis abdominis dorsalis exterior
M l abd ventr: Musculus longitudinalis abdominis ventralis
M t-s: Musculus tergo-sternalis
mg: Midgut
msc-l: Muscle longitudinal
msc-c: Muscle circular
mtub: Malpighian tubules
n: Nucleus
ov: Ovary
ovp: Ovipositor
rER: Rough endoplasmatic reticulum
recp: Rectal papillae
sb: Sperm ball
sb-t: Sperm ball tube
sp: Spermatozoa
sp d I: Spermathecal duct I
sp d II: Spermathecal duct II
sptc: Spermatheca
sptc-L: Spermatheca lumen
sth: Sperm ball sheath
t: Tracheole
v: Vesicle
vag: Vagina
*: Secretion
Arrow: Defective spermatozoa
Arrowhead: Sheath of sperm ball

## References

[1] Cavalleri, A., Masumoto, M., Minaei, K., Mound, L. & Ulitzka, M. R. ThripsWiki - providing information on the World's thrips. https://thrips.info/wiki/Main_Page (2022).

[2] Kirk WDJ, de Kogel WJ, Koschier EH, Teulon DAJ (2021). Semiochemicals for thrips and their use in pest management. Annu. Rev. Entomol..

[3] Mota-Sanchez, D. & Wise, C. J. The Arthropod Pesticide Resistance Database. Available at http://www.pesticideresistance.org (2022). 10.3390/insects15100747 PMC11509053 39452324

[4] von Kèler S (1963). Entomologisches Wörterbuch. mit besonderer Berücksichtigung der morphologischen Terminologie.

[5] Pascini TV, Martins GF (2017). The insect spermatheca: an overview. Zoology Jena (Germany).

[6] Bode, W. Der Ovipositor und die weiblichen Geschlechtswege der Thripiden (Thysanoptera, Terebrantia). *Z. Morph. Tiere (Zeitschrift für Morphologie der Tiere)* **81,** 1–53; 10.1007/BF00290072 (1975).

[7] Moritz, G. Zur Morphologie und Anatomie des Fransenflüglers *Aeolothrips intermedius* Bagnall, 1934 (Aeolothripidae, Thysanoptera, Insecta) III. Mitteilung: Das Abdomen. Investigation on the Morphology and Anatomy in *Aeolothrips intermedius* Bagnall, 1934 (Aeolothripidae, Thysanoptera, Insecta) 3. The Abdomen. *Zoologische Jahrbücher/ Abteilung für Anatomie und Ontogenie der Tiere* **108,** 293–340 (1982).

[8] Moritz, G. Die Ontogenese der Thysanoptera (lnsecta) unter besonderer Berücksichtigung des Fransenflüglers *Hercinothrips femoralis* (O. M. REUTER, 1891) (Thysanoptera, Thripidae, Panchaetothripinae) VI. Imago - Abdomen. The Ontogenesis of Thysanoptera (Insecta) with Special Reference to the Panchaetothripine *Hercinothrips femoralis* (O. M. REUTER, 1891) (Thysanoptera, Thripidae, Panchaetothripinae) VI. Imago - Abdomen. *Zoologische Jahrbücher/ Abteilung für Anatomie und Ontogenie der Tiere* **119,** 157–217 (1989).

[9] Heming BS (1970). Postembryonic development of the female reproductive system in *Frankliniella fusca* (Thripidae) and *Haplothrips verbasci* (Phlaeothripidae) (Thysanoptera). Misc. Publ. Entomol. Soc. Am..

[10] Heming, B. S. History of the germ line in male and female thrips. In *Thrips Biology and Management. International conference on Thysanoptera: Towards Understanding Thrips Management, Vermont,* edited by B. L. Parker, M. Skinner & T. Lewis (Springer, Berlin, 1995), pp. 505–535.

[11] Dallai, R., Del Bene, G. & Lupetti, P. Fine structure of spermatheca and accessory gland of *Frankliniella occidentalis* (Pergande) (Thysanoptera: Thripidae). *Int. J. Insect Morphol. Embryol.* **25,** 317–330; 10.1016/0020-7322(95)00018-6 (1996).

[12] Jordan K (1888). Anatomie und Biologie der Physapoda. Zeitschrift für wissenschaftliche Zoologie.

[13] Bournier, A. L`appareil gènital femelle de *Caudothrips buffai* Karny et sa pompe spermatige (Thysan.). *Bulletin de la Sociètè Entomologique de France* **67,** 203–207 (1962).

[14] Dhileepan K, Ananthakrishnan T (1987). Impact of sex-limited and alary polymorphism on spermathecal diversity and reproductive behaviour in some mycophagous Tubulifera. Proc. Indian Natl. Acad. Sci..

[15] Bhatti JS (1988). The spermatheca as a useful character for species differentiation in *Coleothrips* Haliday (Insecta: Terebrantia: Aeolothripidae). J. Pure Appl. Zool..

[16] Klocke F (1926). Beiträge zur Anatomie und Histologie der Thysanopteren. Zeitschrift für wissenschaftliche Zoologie.

[17] Priesner, H. *Die Thysanopteren Europas* (F. Wagner Verlag, Wien, 1926–1928).

[18] Bode W (1983). Spermienstruktur und Spermatohistogenese bei *Thrips validus* Uzel (Insecta, Thysanoptera). Zoologische Jahrbücher/ Abteilung für Anatomie und Ontogenie der Tiere.

[19] Moritz, G. *Thripse. Fransenflügler, Thysanoptera.* 1st ed. (Westarp Wissenschaften, Hohenwarsleben, 2006).

[20] Bournier A (1956). Contribution à l'étude de la parthénogénèse des thysanoptères et de sa cytologie. Archives de Zoologie expérimentale et générale.

[21] Heming BS (1970). Postembryonic development of the male reproductive system in *Frankliniella fusca* (Thripidae) and *Haplothrips verbasci* (Phlaeothripidae) (Thysanoptera). Misc. Publ. Entomol. Soc. Am..

[22] Krueger, S., Jilge, M., Mound, L. & Moritz, G. B. Reproductive behavior of *Echinothrips americanus* (Thysanoptera: Thripidae). *J. Insect Sci.* **17**; 10.1093/jisesa/iex043 (2017). 10.1093/jisesa/iex043 PMC5469385 28931160

[23] Krueger, S. & Moritz, G. Sperm ultrastructure in arrhenotokous and thelytokous Thysanoptera. *Arthropod Struct. Dev.* **64,** 101084; 10.1016/j.asd.2021.101084 (2021). 10.1016/j.asd.2021.101084 34293581

[24] Lewis, T. *Thrips. Their biology, ecology and economic importance* (Academic Press, London, 1973).

[25] Jacobs W, Seidel F (1975). Systematische Zoologie, Insekten.

[26] Gotoh, A., Ito, F. & Billen, J. Vestigial spermatheca morphology in honeybee workers, *Apis cerana* and *Apis mellifera*, from Japan. *Apidologie* **44,** 133–143; 10.1007/s13592-012-0165-6 (2013).

[27] Gotoh, A., Billen, J., Hashim, R. & Ito, F. Degeneration patterns of the worker spermatheca during morphogenesis in ants (Hymenoptera: Formicidae). *Evol. Dev.* **18,** 96–104; 10.1111/ede.12182 (2016). 10.1111/ede.12182 26994860

[28] Schoeters, E. & Billen, J. The importance of the spermathecal duct in bumblebees. *J. Insect Physiol.* **46,** 1303–1312; 10.1016/S0022-1910(00)00052-4 (2000). 10.1016/S0022-1910(00)00052-4 10844149

[29] Gobin, B., Ito, F., Peeters, C. & Billen, J. Queen-worker differences in spermatheca reservoir of phylogenetically basal ants. *Z. Zellforsch (Zeitschrift für Zellforschung und Mikroskopische Anatomie)* **326,** 169–178; 10.1007/s00441-006-0232-2 (2006). 10.1007/s00441-006-0232-2 16773314

[30] Gobin, B., Ito, F., Billen, J. & Peeters, C. Degeneration of sperm reservoir and the loss of mating ability in worker ants. *Die Naturwissenschaften* **95,** 1041–1048; 10.1007/s00114-008-0420-x (2008). 10.1007/s00114-008-0420-x 18704355

[31] Gotoh, A., Billen, J., Hashim, R., Ito, F. Comparison of spermatheca morphology between reproductive and non-reproductive females in social wasps. *Arthropod. Struct. Dev.* **37,** 199–209; 10.1016/j.asd.2007.11.001 (2008). 10.1016/j.asd.2007.11.001 18342263

[32] Gotoh, A., Billen, J., Tsuji, K., Sasaki, T. & Ito, F. Histological study of the spermatheca in three thelytokous parthenogenetic ant species, *Pristomyrmex punctatus*, *Pyramica membranifera* and *Monomorium triviale* (Hymenoptera: Formicidae). *Acta Zool.* **93,** 200–207; 10.1111/j.1463-6395.2010.00498.x (2012).

[33] Buffa P (1911). Studi intorno al ciclo partenogenetico dell´ *Heliothrips haemorrhoidales* (Boúche). REDIA.

[34] Bene, G. D., Cavallo, V., Lupetti, P. & Dallai, R. Ultrastructure of the accessory gland in the parthenogenetic thrips *Heliothrips haemorrhoidalis* (Bouché) (Thysanoptera. Thripidae). *Int. J. Insect Morphol. Embryol.* **27,** 255–261; 10.1016/S0020-7322(98)00018-X (1998).

[35] Nakao S, Yabu S (1998). Ethological and chemical discrimination between thelytokous and arrhenotokous *Thrips nigropilosus* Uzel, with discussion of taxonomy. Jpn. J. Appl. Entomol. Zool..

[36] Arakaki, N., Miyoshi, T. & Noda, H. Wolbachia-mediated parthenogenesis in the predatory thrips *Franklinothrips vespiformis* (Thysanoptera: Insecta). *Proc. R. Soc. B Biol. Sci.* **268,** 1011–1016; 10.1098/rspb.2001.1628 (2001). 10.1098/rspb.2001.1628 PMC1088702 11375084

[37] Kumm S, Moritz G (2008). First detection of Wolbachia in arrhenotokous populations of thrips species (Thysanoptera: Thripidae and Phlaeothripidae) and its role in reproduction. Environ. Entomol..

[38] Moritz, G. The biology of thrips is not the biology of their adults: a developmental view. In *Thrips and Tospovirus: Proceedings of the 7th International Symposium on Thysanoptera,* edited by L. A. Mound & R. Marullo (Australian National Insect Collection CSIRO, Canberra, 2002), pp. 259–267.

[39] Moritz G, Schäfer E, Kumm S, Steller A, Tschuch GD (2004). Alien-Thrips: *Suocerathrips linguis* - Biologie und Verhalten. Mitteilungen der Deutschen Gesellschaft für allgemeine und angewandte Entomologie.

[40] Gehlsen, U. Ernährungssystem, Verhalten und Wehrsekret des subsozialen Phlaeothripinen Suocerathrips linguis MOUND & MARULLO, 1994 (Insecta, Thysanoptera, Tubulifera). PhD-thesis (Martin-Luther University Halle-Wittenberg, Germany, 2009).

[41] Kumm, S. Reproduction, progenesis and embryogenesis of thrips (Thysanoptera, Insecta). Dissertation. Martin-Luther-Universität Halle-Wittenberg, 2002.

[42] Krueger, S., Mound, L. A. & Moritz, G. B. Offspring sex ratio and development are determined by copulation activity in *Echinothrips americanus* MORGAN 1913 (Thysanoptera: Thripidae). *J. Appl. Entomol.* **140,** 462–473; 10.1111/jen.12280 (2016).

[43] Oetting, R. D., Beshear, R. J., Liu, T.-X., Braman S. K., & Baker, J. R. Biology and identification of thrips on greenhouse ornamentals. *Univ. Ga. Res. Bull.* **414,** 20 (1993).

[44] Mound, L. A., Nielsen, M.-C. & Hastings, A. Thysanoptera Aotearoa. Thrips of New Zealand. Available at https://keys.lucidcentral.org/keys/v3/nz_thrips/index.html.

[45] Schindelin J (2012). Fiji an open-source platform for biological-image analysis. Nat. Methods.

[46] Uzel, H. *Monographie der Ordnung Thysanoptera* (Nabu Oress, Charleston SC, United States, 1895).

[47] Marzo L. De. Dettagli anatomici dei genitali interni in *Melanthrips fuscus* (Sulzer) e altri tisanotteri. *Entomologica* **36,** 109–119. 10.15162/0425-1016/747 (2002).

[48] Melis A (1934). Nuove osservazioni anatomo-istologiche sui diversi stati postembrionali del *Liothrips oleae* Costa. REDIA.

[49] van der Kooi CJ, Schwander T (2014). On the fate of sexual traits under asexuality. Biol. Rev..

[50] Sloan NS, Simmons LW (2019). The evolution of female genitalia. J. Evol. Biol..

[51] Buffa P (1909). Tisanotteri esotici esistenti nel Museo Civico di Storia Naturale di Genova. REDIA.

[52] Osborn H (1895). Note on a New Species of *Phloeothrips*, with description. Proc. Iowa Acad. Sci..

[53] Mound LA, Marullo R (1994). New thrips on mother-in-law`s tongue. Entomol. Mon. Mag..

[54] Zur Strassen, R. *Die terebranten Thysanopteren Europas und des Mittelmeer-Gebietes* (Goecke und Evers, Keltern, 2003).

[55] Davies RG (1961). The postembryonic development of the female reproductive system in *Limothrips cerealium* Haliday (Thysanoptera: Thripidae). Proc. Zool. Soc. Lond..

[56] Gerber GH (1970). Evolution of the methods of spermatophore formation in pterygotan insects. Can. Entomol..

[57] Dallai R, Afzelius BA, Lanzavecchia S, Bellon PL (1991). Bizarre flagellum of thrips spermatozoa (Thysanoptera, Insecta). J. Morphol..

[58] Paccagnini E, Mencarelli C, Mercati D, Afzelius BA, Dallai R (2007). Ultrastructural analysis of the aberrant axoneme morphogenesis in thrips (Thysanoptera, Insecta). Cell Mot. Cytoskel..

[59] Paccagnini E, Lupetti P, Afzelius BA, Dallai R (2009). New findings on sperm ultrastructure in thrips (Thysanoptera, Insecta). Arthropod. Struct. Dev..

[60] Paccagnini E, Mercati D, Giusti F, Conti B, Dallai R (2010). The spermatogenesis and the sperm structure of Terebrantia (Thysanoptera, Insecta). Tissue & cell.

[61] Pitnick S, Wolfner MF, Dorus S (2020). Post-ejaculatory modifications to sperm (PEMS). Biol. Rev. Camb. Philos. Soc..

[62] Karr, T. L., Swanson, W. J. & Snook, R. R. The evolutionary significance of variation in sperm–egg interactions. In *Sperm biology. An evolutionary perspective,* edited by T. R. Birkhead. 1st ed. (Academic Press/Elsevier, Amsterdam, 2009), pp. 305–365.

[63] Friedländer M, Jeshtadi A, Reynolds SE (2001). The structural mechanism of trypsin-induced intrinsic motility in *Manduca sexta* spermatozoa in vitro. J. Insect Physiol..

[64] Hughes M, Davey KG (1969). The activity of spermatozoa of Periplaneta. J. Insect Physiol..

[65] Longo G (1993). Ultrastructural changes in sperm of *Eyprepocnemis plorans* (Charpentier) (Orthoptera: Acrididae) during storage of gametes in female genital tract. Inverteb. Reprod. Dev..

[66] Makielski SK (1966). The structure and maturation of the spermatozoa of *Sciara coprophila*. J. Morphol..

[67] Giuffrida A, Rosati F (1993). Changes in sperm tail of *Eyprepocnemis plorans* (Insects, Orthoptera) as a result of in vitro incubation in spermathecal extract. Inverteb. Reprod. Dev..

[68] Giuffrida A, Focarelli R, Lampariello R, Thole H, Rosati F (1996). Purification and properties of a 35 kDa glycoprotein from spermathecal extract of E*yprepocnemis plorans* (Insecta, Orthoptera) with axonemal cytoskeleton disassembly activity. Insect Biochem. Mol. Biol..

[69] Arakaki N, Noda H, Yamagishi K (2000). Wolbachia-induced parthenogenesis in the egg parasitoid *Telenomus nawai*. Entomol. Exp. Appl..

[70] Pannebakker BA (2005). Sexual functionality of *Leptopilina clavipes* (Hymenoptera: Figitidae) after reversing Wolbachia-induced parthenogenesis. J. Evol. Biol..

[71] Stouthamer R, Russell JE, Vavre F, Nunney L (2010). Intragenomic conflict in populations infected by Parthenogenesis Inducing Wolbachia ends with irreversible loss of sexual reproduction. BMC Evol. Biol..

[72] Sänger K, Helfert B (1994). Comparative studies on number and position of the micropyles and the shape of the eggs of Saga pedo, S natoliae and S ephippigera (Orthoptera: Tettigoniidae). Entomologia.

[73] Gottlieb Y, Zchori-Fein E (2001). Irreversible thelytokous reproduction in *Muscidifurax uniraptor*. Entomol. Exp. Appl..

[74] Stouthamer R, Breeuwer JAJ, Hurst GDD (1999). WOLBACHIA PIPIENTIS. Microbial manipulator of arthropod reproduction. Annu. Rev. Microbiol..

[75] Schwander T, Crespi BJ, Gries R, Gries G (2013). Neutral and selection-driven decay of sexual traits in asexual stick insects. Proc. Biol. Sci..

[76] Werren JH, Baldo L, Clark ME (2008). Wolbachia: master manipulators of invertebrate biology. Nat. Rev. Micro..

