# Supplementary material for: Investigation of the spermathecal morphology, reproductive strategy and fate of stored spermatozoa in three important thysanopteran species
Source: Sci Rep. 2022 Nov 2;12:18517. doi: 10.1038/s41598-022-23104-0 (PMC9630458; doi:10.1038/s41598-022-23104-0)
Supplement: Supplementary file 1 — Supplementary Information 1. [file 41598_2022_23104_MOESM1_ESM.docx]

Supplementary video legend:

Supplementary video 1:

*Suocerathrips linguis* nano-CT scan and volume-rendered 3D-model of the ampoule, transversal and sagittal direction

Supplementary video 2:

*Echinothrips americanus* nano CT scan of the spermatheca with stored spermatozoa in a sperm ball, oblique direction

Supplementary video 3:

*Hercinothrips femoralis* nano CT scan and volume-rendered 3D- model of the spermatheca with stored spermatozoa in a sperm ball, oblique direction
